# Numerical Study on the Coagulation and Breakage of Nanoparticles in the Two-Phase Flow around Cylinders

**DOI:** 10.3390/e24040526

**Published:** 2022-04-08

**Authors:** Ruifang Shi, Jianzhong Lin, Hailin Yang

**Affiliations:** 1 State Key Laboratory of Fluid Power and Mechatronic System, Zhejiang University, Hangzhou 310027, China; 11924031@zju.edu.cn (R.S.); yanghailin@zju.edu.cn (H.Y.); 2 Laboratory of Impact and Safety Engineering, Ningbo University, Ministry of Education, Ningbo 315201, China

**Keywords:** nanoparticle two-phase flow, particle coagulation and breakage, flow around circular cylinders, particle distribution

## Abstract

The Reynolds averaged N-S equation and dynamic equation for nanoparticles are numerically solved in the two-phase flow around cylinders, and the distributions of the concentration *M*_0_ and geometric mean diameter *d_g_* of particles are given. Some of the results are validated by comparing with previous results. The effects of particle coagulation and breakage and the initial particle concentration *m*_00_ and size *d*_0_ on the particle distribution are analyzed. The results show that for the flow around a single cylinder, *M*_0_ is reduced along the flow direction. Placing a cylinder in a uniform flow will promote particle breakage. For the flow around multiple cylinders, the values of *M*_0_ behind the cylinders oscillate along the spanwise direction, and the wake region in the flow direction is shorter than that for the flow around a single cylinder. For the initial monodisperse particles, the values of *d_g_* increase along the flow direction and the effect of particle coagulation is larger than that of particle breakage. The values of *d_g_* fluctuate along the spanwise direction; the closer to the cylinders, the more frequent the fluctuations of *d_g_* values. For the initial polydisperse particles with *d*_0_ = 98 nm and geometric standard deviation *σ* = 1.65, the variations of *d_g_* values along the flow and spanwise directions show the same trend as for the initial monodisperse particles, although the differences are that the values of *d_g_* are almost the same for the cases with and without considering particle breakage, while the distribution of *d_g_* along the spanwise direction is flatter in the case with initial polydisperse particles.

## 1. Introduction

The particle-laden flow around cylinders has attracted the attention of many scholars because of its extensive industrial applications. The particle dispersion and distribution in the wake behind cylinders have been extensively studied in experimental and numerical simulations during the past decade. Zhou et al. [1] showed that the particle distribution was dependent on the Stokes number (*St*). The particles dispersed into the core regions of the vortex at small *St* values concentrated on the boundary of vortex at intermediate *St* values and assembled in the outer region of the vortex at large *St* values. Haddadi et al. [2] found that the hydrodynamic interaction between particles led to the exchange of particles between the wake area and the free stream. Jafari et al. [3] indicated that particle motion in the wake was strongly affected by vortex shedding, while the particle Brownian diffusion influenced the deposition rate of particles. Haddadi et al. [4] studied the suspension around the cylinder with a particle fraction of about 0.08 in the microchannel and found that particles could escape the wake area due to velocity fluctuations by increasing the particle number in the wake. There was also particle exchange between the wake area and the free stream. Huang et al. [5] indicated that the particle collection efficiency was positively related to the particle formation fraction, while the thermophoresis enhanced the impaction efficiency of particles by 1–2 orders. Jeong and Kim [6] indicated that the thermophorectic effect on the particles was obvious at small *St* values, while the deposition efficiency of particles was increased by increasing the temperature difference between the flow and the cylinder or by decreasing the ratio of thermal conductivity of the particle to the fluid. The particles with small *St* values follow the fluid in the upstream surface of the cylinder without collision, but move backward in the downstream direction. Gopan et al. [7] developed correlations for particle temperatures and impaction rates based on the flow and boundary conditions, as well as particle properties.

However, most of the above studies involved large particles, without considering the coagulation and breakage of particle clusters after coagulation. In practical applications, particle coagulation and breakage often occur, which affects the distribution of the particle concentration and sizein the wake behind cylinders. In addition, particle dispersion is dominant when the particle density is low, while it is necessary to consider the coagulation caused by particle collision when the particle density is high. Keita et al. [8] numerically studied the nanoparticle dispersion at *Re* = 9300. They found that the Brownian diffusion tended to concentrate the particles at the edges of the vortices, while the turbulence dispersed particles from the periphery to the core of the vortices. Tu and Zhang [9] experimentally studied the condensation of submicron- and nanoscale particles within *Re* = 5200–35,000. They found that the particle diameter downstream of the cylinder was larger than that upstream, and the total particle concentration and geometric mean diameter in the free stream werelarger and less than in the wake, respectively. Multi-cylinder alignment could be used to enhance particle coagulation. Liu et al. [10] numerically and experimentally studied the structural properties of the vortex generators affecting the particle coagulation. They showed that the optimal efficiency of the particle coagulation was about 16.42% for particles with sizes ranging from 15.7 to 850.0 nm at aflow velocity of 4.8 m/s. Particle collision and coagulation mainly occurred in the windward boundary layer of the vortex generator and at the longitudinal edges of the vortices. Kolsiet al. [11] numerically studied the effects of using double rotating cylinders and partly porous layers in the bifurcating channels on the hydrothermal performance and indicated that the proposed methods of heat transfer enhancement could be considered simultaneously for effective control of the thermal performance of those systems. Alsaberyet al. [12] investigated transient entropy generation and mixed convection due to a rotating hot inner cylinder within a square cavity with a flexible side wall and achieved the highest average heat transfer and global entropy generation rates for counter-clockwise rotation of the circular cylinder and lower values in terms of flexible wall deformation.

From the above, it can be seen that there are few studies on particle distribution while simultaneously considering the effects of particle coagulation and breakage, and research results for the two-phase flow of nanoparticles around multiple cylinders are also rare. In this work, a numerical simulation is carried out to study the distribution of the particle concentration and particle size in the two-phase flow of nanoparticles around a single cylinder and multiple cylinders. The flow of fluid around cylinders is selected because this kind of flow is the most common in practical applications. Meanwhile, the effects of particle coagulation and breakage, initial particle concentration, and size on the particle distribution are discussed.

## 2. Governing Equations

**Figure 1:**
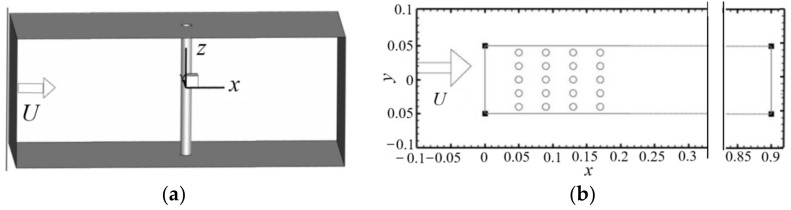
Nanoparticle-laden flows: (**a**) flow around a cylinder; (**b**) flow around 20 cylinders.

For the nanoparticle-laden flow around cylinders, as shown in Figure 1 the distance between two walls in the *z* direction is long enough and there is no velocity in the *z* direction, meaning that the change of the flow along the *z* direction can be ignored. Therefore, the three-dimensional flow can be reduced to flow the in *x*-*y* plane. The distribution of the particle concentration and size in the wake behind cylinders is closely related to the flow characteristics. In Figure 1a, the cylinder diameter is *D* = 50 mm and the geometric center of the cylinder is used as the coordinate origin. Coordinates *x*, *y,* and *z* represent the flow, vertical direction, and span direction, respectively. In Figure 1b, the cylinder diameter is *D* = 10 mm; the flow direction positions of the four columns of cylinders are *x*= 0.05, 0.09, 0.13, and 0.17 m, respectively; and the spanwise positions of the five rows of cylinders are *y* = ±0.02, 0, and ±0.04 m, respectively. The Reynolds number of the flow is defined as *Re* = *UD*/*ν*.

### 2.1. Fluid Flow

For the incompressible flow, the continuity and the Reynolds averaged N-S equations are:

(1) $$\frac{\partial u_{i}}{\partial x_{i}}=0$$

(2) $$\rho \left(\frac{\partial u_{i}}{\partial t}+u_{k}\frac{\partial u_{i}}{\partial x_{k}}\right)=-\frac{\partial p}{\partial x_{i}}+\frac{\partial }{\partial x_{j}}\left(\mu \frac{\partial u_{i}}{\partial x_{j}}\right)+\frac{\partial }{\partial x_{j}}\left(-\rho \bar{u_{i}^{\prime }u_{j}^{\prime }}\right)$$

where *u_i_* is the mean velocity, *ρ* is the density, *p* is the pressure, *μ* is the fluid viscosity, and $\bar{u_{i}{}^{\prime }u_{j}{}^{\prime }}$ is the Reynolds stress, which is modeled by:

(3) $$-\rho \bar{u_{i}{}^{\prime }u_{j}{}^{\prime }}=\rho \nu_{t}\left(\frac{\partial u_{i}}{\partial x_{j}}+\frac{\partial u_{j}}{\partial x_{i}}\right)-\frac{2}{3}\rho k\delta_{ij}$$

in which *ν_t_* = *C_μ_k*^2^/*ε*, where *C_μ_* is a function of the average strain rate, and *k* and *ε* are the turbulent kinetic energy and dissipation rate, respectively. The standard *k*-*ε* turbulence model is selected here because it is suitable for flow with Reynolds numbers in the range of 300 < *Re* < 3 × 10^5^ and is not directly affected by the wall. The transport equations of *k* and *ε* are:

(4) $$\frac{\partial k}{\partial t}+u_{j}\frac{\partial k}{\partial x_{j}}=\frac{\partial }{\partial x_{j}}\left[\left(\nu +\frac{\nu_{t}}{\sigma_{k}}\right)\frac{\partial k}{\partial x_{j}}\right]-\rho_{f}\bar{u_{i}{}^{\prime }u_{j}{}^{\prime }}\frac{\partial u_{i}}{\partial x_{j}}-\varepsilon$$

(5) $$\frac{\partial \varepsilon }{\partial t}+u_{j}\frac{\partial \varepsilon }{\partial x_{j}}=\frac{\partial }{\partial x_{j}}\left[\left(\nu +\frac{\nu_{t}}{\sigma_{\varepsilon }}\right)\frac{\partial \varepsilon }{\partial x_{j}}\right]+\frac{\varepsilon }{k}\nu_{t}C_{\varepsilon 1}\left(-\rho_{f}\bar{u_{i}{}^{\prime }u_{j}{}^{\prime }}\frac{\partial u_{i}}{\partial x_{j}}\right)-\rho_{f}C_{\varepsilon 2}\frac{\varepsilon^{2}}{k}$$

where *C_μ_* = 0.09, *C_ε_*_1_ =1.44, *C_ε_*_2_ =192, *σ_k_* = 1.0, and *σ_ε_* = 1.3.

### 2.2. General Dynamic Equation for Nanoparticles

The instantaneous general dynamic equation for nanoparticles considering the convection, diffusion, and particle coagulation and breakage is [13,14]:

(6) $$\begin{array}{c} \frac{\partial n\left(v,t\right)}{\partial t}+u_{i}\frac{\partial n\left(v,t\right)}{\partial x_{i}}-\frac{\partial }{\partial x_{i}}\left[D_{T}\frac{\partial n\left(v,t\right)}{\partial x_{i}}\right]=\frac{1}{2}\int_{0}^{v}\beta \left(v_{1},v-v_{1}\right)n\left(v_{1},t\right)n\left(v-v_{1},t\right)dv_{1}\\ -\int_{0}^{\infty }\beta \left(v_{1},v\right)n\left(v,t\right)n\left(v_{1},t\right)dv_{1}+\int_{v}^{\infty }a\left(v_{1}\right)b(v|v_{1})n\left(v_{1}\right)dv_{1}-a\left(v\right)n\left(v\right), \end{array}$$

where *n* is the spatial distribution of the number of particles with volume *v* at time *t*, and *D_T_* is the turbulent diffusion coefficient, which is approximated by the turbulent viscosity of the fluid [15]. The first two terms on the right hand side of Equation (6) are the generation and disappearance of particles with volume *v* caused by coagulation, while *β*(*v*_1_,*v*) is the kernel function of coagulation of the two particles with volume *v* and volume *v*_1_ and consists of two parts, i.e., *β* = *β_B_* + *β_T_*, where *β_B_* is the coagulation kernel caused by Brownian motion [16]:

(7) $$\beta_{B}=\frac{2KT}{3\mu }\left(\frac{1}{v^{1/3}}+\frac{1}{v_{1}^{1/3}}\right)\left(v^{1/3}+v_{1}^{1/3}\right)+\frac{2KT}{3\mu }\frac{1.591\lambda }{{\left(3/4\pi \right)}^{1/3}}\left(\frac{1}{v^{2/3}}+\frac{1}{v_{1}^{2/3}}\right)\left(v^{1/3}+v_{1}^{1/3}\right)$$

where *K* is the Boltzmann constant, *T* is the temperature, and *λ* is the average free path of gas molecules. Here, *β_T_* is the coagulation kernel caused by turbulent shear [17]:

(8) $$\beta_{T}=\sqrt{\frac{3}{10\pi }}{\left(v^{1/3}+v_{1}^{1/3}\right)}^{3}{\left(\frac{\varepsilon }{\nu }\right)}^{1/2}$$

The last two terms on the right hand side of Equation (6) are the generation and disappearance of particles caused by breakage. The volume-based breakage kernel function *a*(*v*) represents the breakage frequency of particles with volume *v*. Particle breakage is related to the particle size, velocity gradient, and particle concentration. The exponential breakage kernel function obtained by fitting the experimental data by Spicer [18] is:

(9) $$a\left(v\right)=A{\left(\frac{\varepsilon }{\nu }\right)}^{0.8}v^{1/3}$$

where *A* is 0.47 m^−1^s^−0.6^. In Equation (6), the particle breakage distribution *b*(*v*|*v*_1_) gives the relationship between the parent particles and separated sub-particles. Large particle breakage is not a simple process. The floc is assumed to be composed of small particles of the same size, meaning the distribution function of large, symmetrical broken particles composed of monomers is [19]:

(10) $$b(v|v_{1})=\left\{\begin{array}{c} 2\;v_{1}=2v\\ 0\;\;\mathrm{else} \end{array}\right.$$

### 2.3. Moment Equation of Particle Density

In order to obtain the number density distribution of particles more effectively, the general dynamic equation for nanoparticles is usually transformed into the moment equation of the particle density.

The *k*-th moment of the particle density is defined as:

(11) $$m_{k}=\int_{0}^{\infty }n\left(v,t\right)v^{k}dv$$

Based on Equation (11), Equation (6) can be transformed into a moment equation by multiplying the terms of Equation (6) by *v^k^* and then integrating this over the entire volume distribution:

(12) $$\begin{array}{r} \frac{\partial m_{k}}{\partial t}+u_{i}\frac{\partial m_{k}}{\partial x_{i}}-\frac{\partial }{\partial x_{i}}\left[D_{T}\frac{\partial m_{k}}{\partial x_{i}}\right]=\frac{1}{2}\int_{0}^{\infty }\int_{0}^{\infty }\left[{\left(v+v_{1}\right)}^{k}-v^{k}-v_{1}^{k}\right]\beta \left(v_{1},v\right)n\left(v,t\right)n\left(v_{1},t\right)dvdv_{1}\\ +\int_{0}^{\infty }v^{k}\int_{v}^{\infty }a\left(v_{1}\right)b(v|v_{1})n\left(v_{1}\right)dv_{1}dv-\int_{0}^{\infty }v^{k}a\left(v\right)n\left(v\right)dv \end{array}$$

Substituting Equations (7)–(11) into Equation (12), it can be found that different fractional moments in the equation are difficult to solve. Therefore, the Taylor series expansion technique [20] is used to transform the fractional moment into the moments represented by the first three moments (i.e., 0, 1, 2):

(13) $$\begin{array}{l} \frac{\partial m_{0}}{\partial t}+u_{i}\frac{\partial m_{0}}{\partial x_{i}}-\frac{\partial }{\partial x_{i}}\left(D_{T}\frac{\partial m_{0}}{\partial x_{i}}\right)=-\sqrt{\frac{3}{10\pi }\frac{\varepsilon }{\nu }}\frac{m_{0}^{3}m_{2}^{2}-20m_{0}^{3}m_{1}^{2}m_{2}+127m_{0}m_{1}^{4}}{27m_{1}^{3}}\\ -0.47{\left(\frac{\varepsilon }{\nu }\right)}^{0.8}\frac{m_{0}^{5/3}m_{2}-10m_{0}^{2/3}m_{1}^{2}}{9m_{1}^{5/3}}+\frac{2kT}{3\mu }\left[\frac{2m_{0}^{4}m_{2}^{2}-13m_{0}^{3}m_{1}^{2}m_{2}-151m_{0}^{2}m_{1}^{4}}{81m_{1}^{4}}\right.\\ \left.-\frac{1.591\lambda }{{\left(3/4\pi \right)}^{1/3}}\frac{-5m_{0}^{13/3}m_{2}^{2}+64m_{0}^{10/3}m_{1}^{2}m_{2}+103m_{0}^{10/3}m_{1}^{4}}{81m_{1}^{13/3}}\right] \end{array}$$

(14) $$\frac{\partial m_{1}}{\partial t}+u_{i}\frac{\partial m_{1}}{\partial x_{i}}-\frac{\partial }{\partial x_{i}}\left(D_{T}\frac{\partial m_{1}}{\partial x_{i}}\right)=0$$

(15) $$\begin{array}{l} \frac{\partial m_{2}}{\partial t}+u_{i}\frac{\partial m_{2}}{\partial x_{i}}-\frac{\partial }{\partial x_{i}}\left(D_{T}\frac{\partial m_{2}}{\partial x_{i}}\right)=-\sqrt{\frac{3}{10\pi }\frac{\varepsilon }{\nu }}\frac{4\left(5m_{0}^{2}m_{2}^{2}+14m_{1}^{4}+35m_{0}m_{1}^{2}m_{2}\right)}{27m_{0}m_{1}}\\ -0.47{\left(\frac{\varepsilon }{\nu }\right)}^{0.8}\frac{14m_{0}m_{1}^{1/3}m_{2}-5m_{1}^{7/3}}{18m_{0}^{4/3}}+\frac{4kT}{3\mu }\left[\frac{2m_{0}^{4}m_{2}^{2}-13m_{0}m_{1}^{2}m_{2}-151m_{1}^{4}}{81m_{1}^{2}}\right.\\ \left.+\frac{1.591\lambda }{{\left(3/4\pi \right)}^{1/3}}\frac{-4m_{0}^{7/3}m_{2}^{2}+8m_{0}^{4/3}m_{1}^{2}m_{2}+320m_{0}^{1/3}m_{1}^{4}}{81m_{1}^{7/3}}\right] \end{array}$$

The zero-order moment *m*_0_ represents the total number of particles of all sizes in the unit volume at a given position and time, which is also called the particle concentration. The first-order moment *m*_1_ represents the volumes of all particles in the unit volume at a given position and time, which is also called the volume concentration. The second-order moment *m*_2_ is related to the dispersion of particles. In the simulation, the dimensionless quantity is defined as *M_n_* = *m_n_*/*m_nn_*(*n* = 0, 1, 2), where *m_nn_* is the initial value of *m_n_*. The geometric mean diameter *d_g_* of particles is defined as:

(16) $$d_{g}={\left(\frac{m_{1}^{2}}{\sqrt{m_{0}^{3}m_{2}}}\right)}^{1/3}$$

## 3. Numerical Simulation

All simulations are based on the finite volume method and are carried out using the OpenFOAM platform. OpenFOAM is an open source CFD software and has an extensive range of features to solve anything from complex fluid flows involving turbulence and heat transfer, to solid mechanics and electromagnetic simulations. The equations of fluid flow are solved numerically with the basic solver pisoFoam in the platform. The piso algorithm takes an iterative approach to deal with the coupled pressure–velocity. The discrete and solution modes of the specific equation terms are also given. Gaussian linear interpolation scheme is employed to discretize divergence terms, gradient terms, and Laplace terms of the equations (interpolated from the body center of the grid unit to the surface center). The equations for nanoparticles are solved numerically with the self- made solver. The one-way coupling method is used, i.e., where the effect of particles on the flow is ignored. The particles are obtained from lit permethrin-based mosquito coils. The particles have a density of 730 kg/m^3^ and a diameter of 98 nm, which are the typical density and diameter of nanoparticles.

### 3.1. Main Steps

The calculation domain is shown in Figure 1, where the boundary conditions are as follows. The inlet velocity is evenly distributed and equal to 2.664 m/s, the pressure boundary condition is adopted at the outlet, and the no slip boundary condition is adopted on the wall. In the simulation, the range of Reynolds numbers is 300 < *Re* < 3 × 10^5^. In this Reynolds number range, the boundary layer on the cylinder surface is laminar but the flow behind the cylinder is turbulent. In addition, the flow around a cylinder with this range of Reynolds number is very common. In the simulation, the grid around a single cylinder is shown in Table 1, where 2D and 3D represent two and three dimension, respectively. Since all boundaries are far enough from the wake region, the effects of the existence of boundaries (i.e., size of the domain) on the solution accuracy can be ignored.

In the simulation, the time step Δ*T* is 1.5 × 10^−4^ s and the courant number is less than 1 for achieving numerical stability and accuracy. The tolerance set by the solver was 10^−5^. The mean velocity and pressure are calculated by adding the field average functions to the control file.

### 3.2. Grid Independence Test and Validation

**Figure 2:**
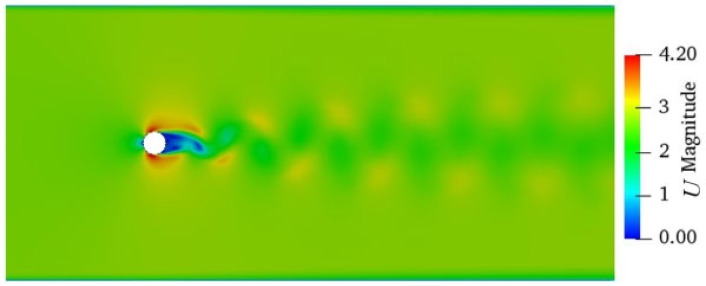
Velocity distribution of flow around a cylinder.

**Figure 3:**
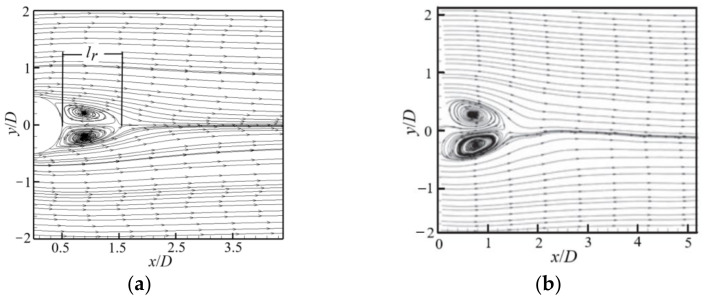
Distribution of time-averaged streamlines: (**a**) present result; (**b**) experimental result [9].

The velocity distribution of the flow around a cylinder is shown in Figure 2, where a stagnation point is formed at the front end of the cylinder. The region close to the tail of the cylinder is the wake region, where the velocity is very small. Downstream of cylinder, two alternating vortices obviously appear. In order to validate the numerical method and code, the present numerical result for the time-averaged streamline in 3D flow is compared with the experimental ones [9], as shown in Figure 3, where both results are in good agreement.

**Figure 4:**
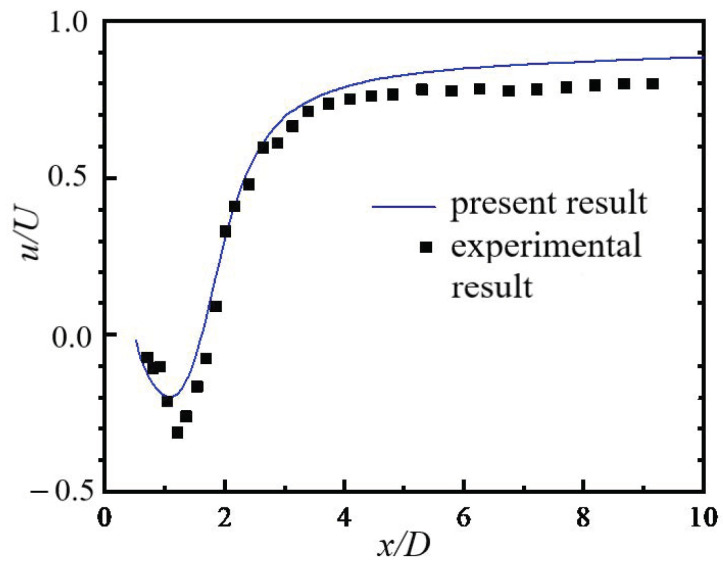
Comparison of streamwise velocity at the centerline.

An important parameter to describe the flow around a cylinder is the reflux length l_r_, which is defined as shown in Figure 3a. The present numerical result for the reflux length l_r_ is further compared with other numerical results. For the present result in 3D flow, l_r_ = 0.08 − 0.025 m = 0.55 m and the particle diameter D = 0.5 m, so l_r_ = 1.1D (*Re* = 9000), as shown in Figure 4. Keita et al. [8] also numerically simulated the flow around a 2D cylinder at *Re* = 9300 and gave a reflux length l_r_ = 0.9D. The main reason for the slight deviation in the value of the reflux length is the difference between the Reynolds number and calculation dimension. Figure 4 shows a comparison of the streamwise velocity at the centerline along the x direction, where it can be seen that the present numerical results are in good agreement with the experimental results [21].

**Figure 5:**
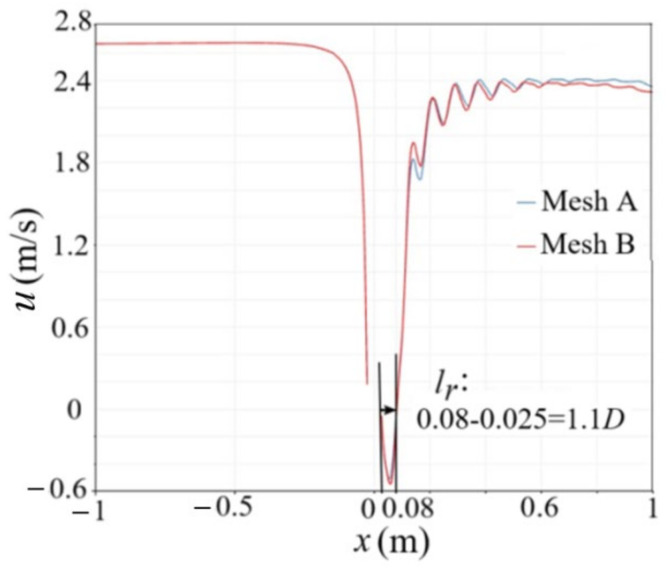
Streamwise velocity at the centerline along the x direction.

A grid independence test is performed by changing the grid number, as shown in Figure 5, where mesh A and mesh B correspond to 28,800 and 46,000 grid numbers, respectively, as shown in Table 1. The results are almost the same for both grid numbers, so mesh A is selected in the simulation.

## 4. Results and Discussion

### 4.1. The Flow around a Single Cylinder

#### 4.1.1. Particle Coagulation and Distribution of Particle Concentration

**Figure 6:**
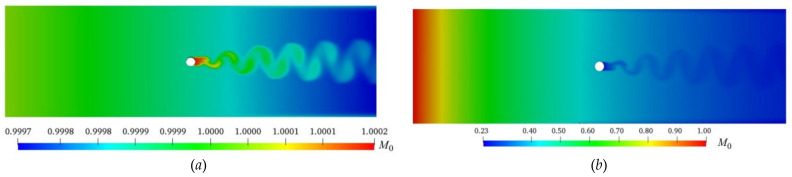
Distribution of *M*_0_ values (*d*_0_ = 98 nm, *Re* = 9000): (**a**) m_00_ = 3.6 × 10^11^/m^3^; (**b**) m_00_ = 3.6 × 10^15^/m^3^.

In a fully developed turbulent wake flow, the particle coagulation mainly results from the Brownian motion and turbulent shear in the free molecular region. The coagulated particles may break up under the action of turbulent shear. The characteristic times for flow convection, particle coagulation, and particle breakage are different. Previous research results have shown that particle coagulation occurs when the ratio of the characteristic time of flow convection to that of particle coagulation is less than 0.1 [22,23,24]. Figure 6 shows the distributions of particle concentration *M*_0_ (=*m*_0_/*m*_00_) with different initial particle concentrations *m*_00_. It can be seen that the values of *M*_0_ upstream of the cylinder are uniformly distributed along the spanwise direction and gradually reduced along the flow direction, which indicates that coagulation has occurred in the process of particle transportation downstream. The values of *M*_0_ are large (as shown in Figure 6a) and small (as shown in Figure 6b) in the wake region close to the tail of the cylinder, indicating that it is easy for the particles with an initial *m*_00_ = 3.6 × 10^11^/m^3^(i.e., relatively low initial particle concentration) to enter the wake area behind the cylinder, although the opposite is true for the particles with an initial *m*_00_ = 3.6 × 10^15^/m^3^. The variation ranges of *M*_0_ are 0.9997~1.0002, as shown in Figure 6a, and 0.23~1.0, as shown in Figure 6b, showing that it is easier for the particles to coagulate when *m*_00_ is high, resulting in a wide distribution range of *M*_0_. An oscillating wake is formed behind the cylinder and the values of *M*_0_ in the oscillating wake are obviously larger (as shown in Figure 6a) and smaller (as shown in Figure 6b) than that around the wake. At the position close to the outlet, the values of *M*_0_ return to the uniform distribution along the spanwise direction. Three conclusions can be drawn from Figure 6: (1) there is an obvious coagulation phenomenon of particles forthe parameters of *m*_00_, *d*_0_, and *Re* given in the paper, and the larger the value of *m*_00_, the larger the differences in values of *M*_0_ in different regions of the flow; (2) the existence of the cylinder has a great influence on the distribution of the particles; (3) *m*_00_ will affect the number of particles in the cylindrical wake.

#### 4.1.2. Function of Particle Breakage

**Figure 7:**
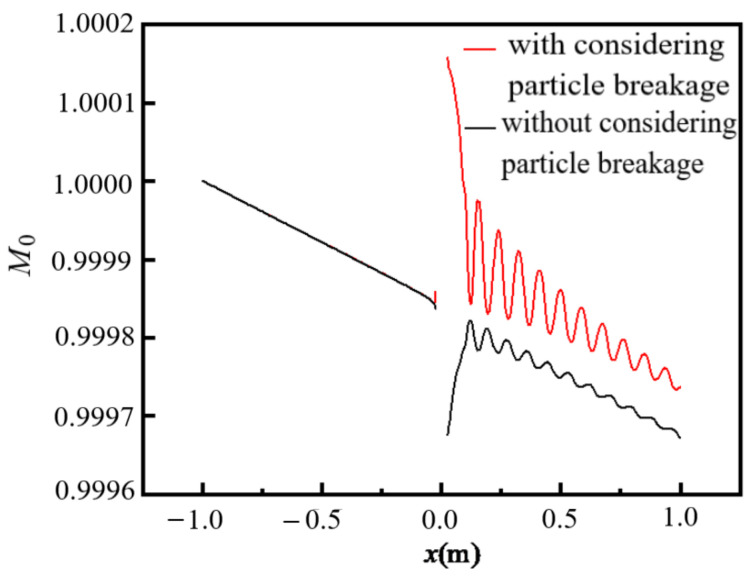
Variations in *M*_0_ at the centerline along the flow direction (*Re* = 9000, *d*_0_ = 98 nm, *m*_00_ = 3.6 × 10^11^/m^3^).

The coagulated particles may break up under the action of turbulent shear. However, whether the coagulated particles are broken depends on the size of coagulated particles and the shear rate of the flow. The variations in particle concentration *M*_0_ (=*m*_0_/*m*_00_) at the centerline along the flow direction are shown in Figure 7. In the flow upstream of the cylinder (*x* < 0), the values of *M*_0_ are decreased along the flow direction due to the occurrence of particle coagulation, and there is almost no difference in *M*_0_ between the cases with and without considering particle breakage because the particles do not have enough time to break up after coagulation and the shear rate of the flow is very small. The discontinuous part of *M*_0_ is located at the position of the cylinder (*x* = 0). In the flow downstream of the cylinder, the values of *M*_0_ for the case considering particle breakage are obviously larger than that for the case without considering particle breakage because the shear rate of the flow downstream of the cylinder is large. A large shear rate is more likely to lead to particle breakage and an increase in *M*_0_. The fluctuation curve shows that the values of *M*_0_ are affected by the wake flow. Therefore, putting an obstacle in a uniform flow will promote particle breakage under a certain particle concentration.

#### 4.1.3. Distribution of Particles along the Spanwise Direction

**Figure 8:**
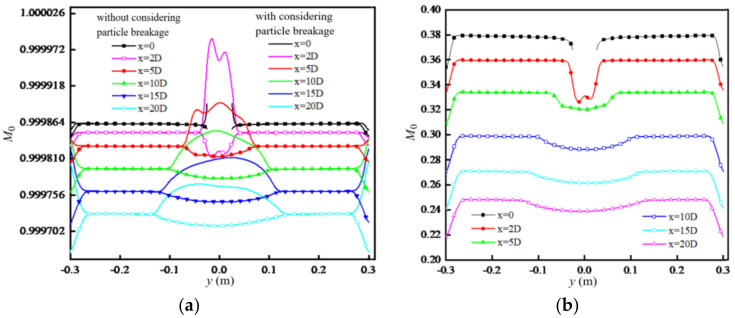
Distribution of *M*_0_: (**a**) m_00_ = 3.6 × 10^11^/m^3^; (**b**) m_00_ = 3.6 × 10^15^/m^3^ (considering particle breakage).

Figure 8 shows the distribution of *M*_0_ along the spanwise direction in the flow downstream of the cylinder for two different *m*_00_. In Figure 8a, the values of *M*_0_ for both cases with and without considering particle breakage decrease along the flow direction, showing that the particle coagulation effect is larger than the breakage effect. The values of *M*_0_ along the spanwise direction, except for the wake regions behind the cylinder and the near wall region, are uniformly distributed and the same for the cases with and without considering particle breakage because the shear rate of the flow is very small in this region.

In the wake region behind the cylinder, the values of *M*_0_ for the case considering particle breakage are obviously larger than that for the case without considering particle breakage becausethe shear rate in this regionis large. As the flow develops downstream, the values of *M*_0_ for both cases with and without considering particle breakage tend to be uniformly distributed along the spanwise direction. Figure 8b shows the distribution of *M*_0_ when considering particle breakage in the case of higher *m*_00_. It can be seen that the distribution of *M*_0_ is qualitatively consistent with that in Figure 8a, but the magnitude of *M*_0_ is far less than that in Figure 8a. The reason is that the higher *m*_00_ increases the chance of particle coagulation, so the value of *M*_0_ (=*m*_0_/*m*_00_) is far less than *m*_00_.

### 4.2. The Flow around Multiple Cylinders

#### 4.2.1. Particle Coagulation and Distribution of Particles

**Figure 9:**
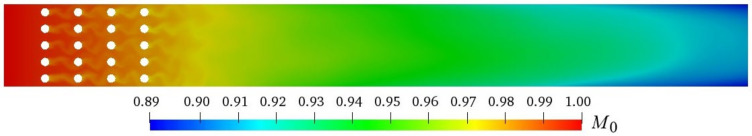
Distribution of *M*_0_ in the flow around multiple cylinders (*d*_0_ = 98 nm, *m*_00_ = 3.6 × 10^14^/m^3^, *Re* = 9000).

For the flow around multiple cylinders, the distribution of *M*_0_ is shown in Figure 9. It can be seen that the values of *M*_0_ upstream of the cylinders are uniformly distributed along the spanwise direction and gradually reduced along the flow direction because of particle coagulation. The values of *M*_0_ behind the cylinders oscillate laterally under the influence of the flow structure in the wake of each cylinder. Compared with the flow around a single cylinder, as shown in Figure 5, the wake region of the flow around multiple cylinders is shorter along the flow direction due to the mutual interference of wakes behind multiple cylinders. The distribution of *M*_0_ along the spanwise direction becomes uniform as the influence of the wake disappears, and turns to a parabolic distribution due to the increase in wall influence at the position close to the outlet.

#### 4.2.2. Distribution of the Geometric Mean Diameter of Particles with the Initial Monodispersity

**Figure 10:**
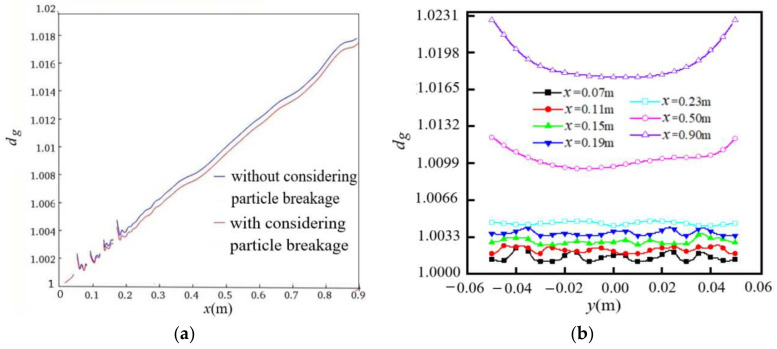
Distribution of *d_g_* for initial monodisperse particles (*d*_0_ = 98 nm, *m*_00_ = 3.6×10^14^/m^3^, *Re* = 9000): (**a**) along the flow direction; (**b**) along the spanwise direction (considering particle breakage).

Figure 10 shows the distribution of the geometric mean diameters *d_g_* of particles along the flow and spanwise directions for initial monodisperse particles. The values of *d_g_* are given along the centerline of the flow in Figure 10a, where there are four jumps and discontinuities in the values of *d_g_* at the positions of the four columns of cylinders. The values of *d_g_* increase along the flow direction because of the occurrence of particle coagulation in the process of particle transportation downstream, showing that the characteristic time of the flow convection is longer than the characteristic time of the particle coagulation and that the particles have enough time to coagulate, while at the same time showing that the effect of particle coagulation is larger than that of particle breakage. The values of *d_g_* are larger for the case without considering particle breakage than that when considering particle breakage, which is reasonable because the number of small particles increases when considering particle breakage. Figure 10b shows the distribution of *d_g_* along the spanwise direction at different positions of *x*. The values of *d_g_* increase along the flow direction and fluctuate along the spanwise direction. The closer to the cylinders, the more frequent the fluctuations of *d_g_* due to the influence of the wake. In the far downstream area (*x* = 0.90), the distribution of *d_g_* along the spanwise direction shows a single arc due to the influence of the boundary. The values of *d_g_* are small in the middle and large on both sides at *x* = 0.50 and *x* = 0.90, which is the reason that the breakage of coagulated particles is larger in the middle area with high *M*_0_.

#### 4.2.3. Distribution of Geometric Mean Diameter of Particles with Initial Polydispersity

**Figure 11:**
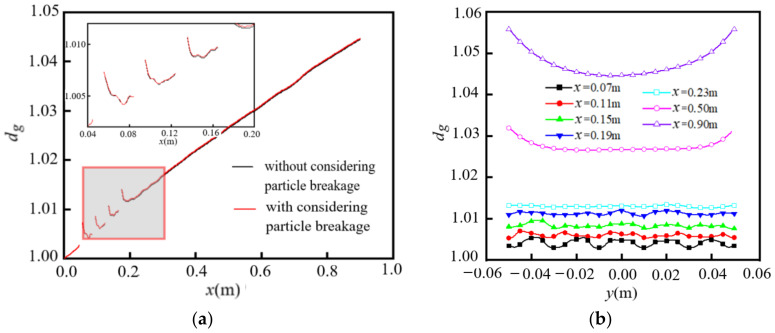
Distribution of *d_g_* for initial polydisperse particles (*d*_0_ = 98 nm, *m*_00_ = 3.6 × 10^14^/m^3^, *Re* = 9000): (**a**) along theflow direction; (**b**) along the spanwise direction (considering particle breakage).

Figure 11 shows the distribution of the geometric mean diameter *d_g_* of particles along the flow and spanwise directions for initial polydisperse particles with *d*_0_ = 98 nm and geometric standard deviation *σ* = 1.65. In Figure 11a, the variations of *d_g_* along the flow direction show the same trend as in Figure 10a, although the difference is that the values of *d_g_* are almost the same for the cases with and without considering particle breakage for the initial polydisperse particles. This is because the particle breakage distribution *b*(*v*|*v*_1_) included in Equation (6) is only for the parent particles composed of separated sub-particles, with the same size as that shown in Equation (10). The particle breakage is insignificant for the polydisperse particles of different sizes. In Figure 11b, the distribution of *d_g_* along the spanwise direction at different positions of *x* is similar to that shown in Figure 10b; the difference is that the distribution of *d_g_* along the spanwise direction is flatter in Figure 11b than in Figure 10b.

## 5. Conclusions

In this paper, the Reynolds averaged N-S equation and general dynamic equation for nanoparticles are numerically solved in the two-phase flow around a single cylinder and multiple cylinders. The distributions of *M*_0_ and *d_g_* values of particles with different *m*_00_ and *d*_0_ values are given. Some of the results are validated by comparing them with the experimental and numerical results. The effects of particle coagulation and breakage, *m*_00_ and *d*_0_, on the particle distribution are discussed. The main conclusions are summarized as follows:

(1) For the flow around a single cylinder, there is an obvious particle coagulation phenomenon. The existence of a single cylinder has a great influence on the distribution of particles. The number of particles in the wake is dependent on the value of *m*_00_. In the flow upstream of the cylinder, there is almost no difference in *M*_0_ between the cases with and without considering particle breakage. Putting a cylinder in a uniform flow will promote particle breakage. As the flow develops downstream, the values of *M*_0_ for both cases with and without considering particle breakage tend to be uniformly distributed along the spanwise direction;
(2) For the flow around multiple cylinders, the values of *M*_0_ are reduced along the flow direction upstream of the cylinders and oscillate laterally behind the cylinders under the influence of the flow structure. For the initial monodisperse particles, the effect of particle coagulation is larger than that of particle breakage. For the initial polydisperse particles with *d*_0_ = 98 nm and geometric standard deviation *σ* = 1.65, the variations in *d_g_* show the same trend as for the initial monodisperse particles, but the differences are that the values of *d_g_* are almost the same for the cases with and without considering particle breakage;
(3) In future work, it will be necessary to further study the numerical simulation of three-dimensional flow and to explore the particle breakage model in the case of polydisperse particles.

## Figures and Tables

**Table 1:** Mesh characteristics.

Case	Flow	Grid Number
A	2D	28,800
B	2D	46,000
C	3D	668,000
